# Neonatal eye screening for 203 healthy term new-borns using a wide-field digital retinal imaging system

**DOI:** 10.1186/s12886-021-01882-x

**Published:** 2021-03-09

**Authors:** Kenneth Teow Kheng Leong, Siti Nur Amira Abu Kassim, Jasvinjeet Kaur Sidhu, Zayani Zohari, Thivakar Sivalingam, Sunder Ramasamy, Safinaz Mohd Khialdin, Noraihan Mohd Nordin, Jamalia Rahmat

**Affiliations:** 1 Department of Ophthalmology, Universiti Kebangsaan Malaysia Medical Centre, Jalan Yaacob Latif, Bandar Tun Razak, 56000 Cheras, Kuala Lumpur Malaysia; 2 Department of Ophthalmology, Hospital Kuala Lumpur, 50586 Jalan Pahang, Kuala Lumpur Malaysia; 3 Department of Obstetrics & Gynaecology, Hospital Kuala Lumpur, 50586 Jalan Pahang, Kuala Lumpur Malaysia

**Keywords:** Healthy, Term, New-born, Universal, Eye screening, Haemorrhage

## Abstract

**Background:**

The current practice for new-born eye examination by an Ophthalmologist in Malaysian hospitals is limited to only preterm new-borns, syndromic or ill infants. Healthy term new-borns are usually discharged without a thorough eye examination. This study is aimed at determining the proportion and types of ocular abnormalities detected in purportedly healthy term new-borns.

**Method:**

This cross-sectional study is comprised of 203 participants, all purportedly healthy term new-born infants from the Obstetrics and Gynaecology ward at Hospital Kuala Lumpur over a 6 months period. The examination list includes external eye examination, red reflex test, and fundus imaging using a wide-field digital retinal imaging system (Phoenix Clinical ICON Paediatric Retinal Camera) by a trained Investigator. The pathologies detected were documented. The results were compared and correlated with similar studies published in the literature previously.

**Results:**

Total ocular abnormalities were detected in 34% of the infants. The most common finding was retinal haemorrhage in 29.6% of the infants, of which 53.3% occurred bilaterally. Spontaneous vaginal delivery (SVD) remained the greatest risk factor which has nearly 3.5 times higher risk of new-borns developing retinal haemorrhage compared to Lower Segment Caesarean Section (LSCS). There was a 6% increased likelihood of developing retinal haemorrhage for every 1-min increment in the duration of 2nd stage of labour.

**Conclusion:**

Universal eye screening for all new-borns using a wide-field digital imaging system is realistically possible, safe, and useful in detecting posterior segment disorders. The most common abnormality detected is retinal haemorrhage.

## Background

The current practice for new-born eye examination by an Ophthalmologist in Malaysian hospitals includes only those deemed at risk. Normal healthy term new-borns will undergo a general examination by the Paediatrician using direct ophthalmoscope by looking at the red reflex and referred to an Ophthalmologist only if those basic screening tests are grossly abnormal. Although the red reflex tests may be quick and simple to perform, a study by *Ming Sun* et al. reported that the proportion of abnormalities that were correctly classified by the red reflex test was better for the anterior segment group (sensitivity = 99.6%) rather than the posterior segment group (sensitivity = 4.1%) [1]. Therefore, the red reflex test is a useful universal screening tool in the detection of anterior abnormalities. However, the test has limitations in detection of posterior abnormalities. This stresses the point that the current eye assessment protocol is insufficient and may in fact have underdiagnosed certain posterior segment eye diseases. However, the main reason why neonatal eye screening is not compulsory for normal term new-borns can be attributed to lack of human resources, time and financial cost.

A few large studies in India and China found that abnormal ocular findings in this group of new-borns ranged from 4 to 25% [2–4]. A pilot study screening 481 older infants at 6 weeks of age in China found up to 41.2% abnormal ocular findings [5]. Abnormal ocular findings detected included subconjunctival haemorrhage, congenital microphthalmos, congenital corneal leukoma, posterior synechia, persistent pupillary membrane, congenital cataract, retinoblastoma, optic nerve defects, vitreous haemorrhage, congenital glaucoma, uveal coloboma and so on [2, 3]. Some of these findings are diseases that may require urgent investigation and early treatment [6].

In 2003, a pilot study first introduced the use of retinal fundus imaging and telemedicine in the approach for the screening of retinopathy of prematurity [7]. However, this approach was limited to premature new-borns. Over the past 5 years, universal new-born’s eye screening is gaining interest as a potential way to reduce long-term visual impairment through early detection and treatment of ocular abnormalities. Several studies in India, China and the United States have taken advantage of the advances in retinal fundus imaging and telemedicine for this purpose [3, 4, 8]. These studies are advocating universal eye screening for all new-borns and *Vinekar* et al. has shown that wide-field digital imaging is possible and safe [4]. These infants showed less pain and stress at 30 s after completing examination to those examined with a binocular indirect ophthalmoscope [9] and the sensitivity and specificity of wide-field digital retinal image (WFDRI) acquisition system is excellent as a fundus screening tool [10].

Given the above, we designed our study using a WFDRI acquisition system device to examine healthy term new-borns to determine the proportion and types of ocular abnormalities, which would have been missed. We hope to improve awareness of ocular abnormalities amongst healthy new-borns and wish to emphasize the importance of universal new-born eye screening.

## Materials and methods

### Subject selection

This is a cross-sectional study of new-borns delivered in the Obstetrics & Gynaecology Labour Room, Hospital Kuala Lumpur (HKL) in Malaysia from January 2019 to June 2019. Healthy term infants selected underwent an ocular examination within the first 72 h or prior discharge. Subjects were enrolled based on a convenience sampling method. Any mother available, who has had delivered and fulfilled the inclusion and exclusion criteria was invited to participate in this study. No attempt was made to contact those mothers who had been discharged home.

The inclusion criteria included neonate (age < 28 days), born term (between 37 weeks, 0 days and 41 weeks, 6 days), birth weight (≥2000 g), APGAR score ≥ 7, any mode of delivery (e.g. spontaneous vaginal delivery, assisted birth and caesarean section), stable vital signs, informed consent given by parent(s) for participation and able to comply to all requirements of the study protocol. Exclusion criteria include born pre-term, dysmorphic babies, syndromic babies, congenital diseases, complications intra & postpartum requiring intensive care and monitoring and unstable for an eye examination.

All subjects who conform to these criteria undergo a non-invasive eye examination, which consisted of a 2-part examination. The first part of the examination examined the anterior segment of the eyes followed by an examination of the posterior segment of the eyes after pupillary dilation. All of these was done at the same setting. The ocular examination included using a direct retinoscope and a WFDRI acquisition system.

Part 1 examined the undilated eye including the red reflex test, external inspection and pupil examination. The new-borns were brought to the examination room in the postnatal ward. An assistant nurse will hold the new-born securely on the examination table throughout the examination. Topical anaesthetic eye drops Tetracaine Hydrochloride 0.5% (Amethocaine®, Zulat Pharmacy Sdn Bhd, China) was applied to anaesthetise the eyes. Eye speculums were gently inserted, and room light was dimmed when the examination was conducted. Bilateral eye anterior segment, pupillary light reflex and red reflex were examined using a direct ophthalmoscope. Image of the anterior segment were captured with a wide-field digital retinal imaging system (Phoenix Clinical ICON Paediatric Retinal Camera®, Phoenix Technology Group Company, CA, USA).

Part 2 examined the fundus of the eyes after dilatation. A mixture of Phenylephrine hydrochloride 2.5% (Mydfrin® 2.5%, Alcon Laboratories Inc., Texas, USA) and Tropicamide 1% (Mydriacyl® 1% Alcon Laboratories Inc., Texas, USA) eye drops were instilled onto the eyes at 10 min interval up to 30 min. After a minimum of 6-8 mm dilation was achieved, the new-borns were brought back into the examination room. An assistant nurse will hold the new-born. Amethocaine was instilled and eye speculum was inserted again. Coupling gel was inserted into the eyes before resting the camera probe on to the eye. A video of the fundus was recorded using the ICON Paediatric Retinal Camera and still images were captured for analysis and record. Five fundus photographs were taken, and they included the posterior pole (including disc and fovea), superior retina, inferior retina, temporal retina and nasal retina. Prophylactic topical antibiotic Fusidic acid 1% (Fucithalmic® 1% LEO Laboratories Limited, Dublin 12, Ireland) ointment was instilled onto both eyes after the procedure.

Ocular examinations and all imaging was done by an Investigator who is an Ophthalmology Medical Officer with more than 5 years’ experience. He is fully trained to use the medical instruments and is aware of all safety precautions while handling these babies. The babies were then monitored by a standby nurse. All photos recorded in the study were further re-evaluated by a Paediatric Ophthalmologist. Any abnormality detected was referred for further management and treatment.

## Statistical analysis

### Formulae for sample size calculation

$$ Sample\ size\ (n)=\frac{{\left({Z}_{1-\frac{\alpha }{2}}\right)}^2\ p\left(1-p\right)}{d^2} $$

Where: n = sample size,

Z = level of confidence,

α = alpha,

p = expected prevalence or proportion, and.

d = precision.

#### With finite population correction

The sample size calculation was based on a pilot study by *Goyal P.* et al. Outcome of universal new-born eye screening with WFDRI acquisition system: a pilot study. Eye (Lond). 2017 Jul [2];.

Labour room census in HKL recorded an estimated 30 deliveries per day or 1000 deliveries per month. Therefore, we estimated a total of 12,000 deliveries per year in HKL. Sample size estimation was calculated using the population proportion formulae (Lemeshow, Hosmer, Klar, Lwanga & Organization, 1990). Prior data indicated that the proportion of ocular abnormality in *Goyal P.* et al. is 0.149 and population size is 12,000. If the Type 1 error probability and precision are 0.05 and 0.05, we will need to study 192 samples. Assuming a drop-out rate of 10%, we require a total of 212 participants.

### Statistical analysis

Data was explored and analysed using SPSS software version 21.0. Numerical variables was presented using mean and standard deviation. Categorical variables was presented as frequency and percentage. Distributions of continuous variables between new-borns with and without ocular abnormalities was compared using Student’s t tests; Pearson’s chi-square tests or Fisher’s exact tests were used for distributions of categorical variables. Statistical significance was defined by a *P* value of less than 0.05. Simple Logistic Regression and Multiple Logistic Regression was used to determine the risk factors associated with ocular abnormalities among healthy term new-born, without or with adjustment of co-variates. All odd ratios (ORs) were presented with 95% confidence intervals (CI).

## Results

Of the 203 new-borns-study who had a complete eye examination, there were 69 new-borns found to have ocular abnormality. The proportion of ocular abnormalities was 34%. The distribution of demographic characteristics of 203 new-borns with and without ocular abnormalities will be shown in Table 1.

**Table 1:** Distribution of demographics of normal and abnormal ocular findings among healthy term new-borns in Hospital Kuala Lumpur

	Normal Ocular Findings		Abnormal Ocular Findings		***P*** value
	***N*** = 134		***N*** = 69		
	Mean (SD)	n (%)	Mean (SD)	n (%)	
**New-borns age (day of life)**	1.94 (1.72)		1.90 (0.89)		0.850 ^a^
**Birth weight (g)**	2991.34 (427.41)		2998.07 (441.90)		0.916 ^a^
**Term**					
37 wks to 38 wks 6 days		82 (61.2)		40 (58.0)	0.879 ^c^
39 wks to 40 wks 6 days		49 (36.6)		27 (39.1)	
41 wks to 41 wks 6 days		3 (2.2)		2 (2.9)	
**Gender**					
Male		78 (58.2)		43 (62.3)	0.572 ^b^
Female		56 (41.8)		26 (37.7)	
**Race**					
Malay		109 (81.3)		50 (72.5)	0.384 ^c^
Chinese		7 (5.2)		6 (8.7)	
Indian		13 (9.7)		11 (15.9)	
Others		5 (3.7)		2 (2.9)	
**Maternal age (year)**	28.73 (6.32)		28.45 (5.68)		0.756 ^a^
**Maternal gravida**					
One		38 (28.4)		22 (31.9)	0.911 ^b^
Two		30 (22.4)		13 (18.8)	
Three		30 (22.4)		13 (18.8)	
Four		20 (14.9)		11 (15.9)	
Five or more		16 (11.9)		10 (14.5)	
**Mode of delivery**					
SVD		64 (47.8)		52 (75.4)	< 0.001 ^b^
LSCS		70 (52.2)		17 (24.6)	

^a^Student’s t test

^b^Pearson’s Chi-Square test

^c^Fischer’s Exact test

The mean new-borns’ age was 1.93 ± 1.49 days of life during an eye examination. There was no significant difference in new-borns’ weight between the group with and without ocular abnormalities. The mean weight was 2993.63 ± 431.31 g. There was no significant difference in maternal age among these two groups. The average maternal age was around 28.64 ± 6.10 years old. There was also no significant difference in terms of gender, race, maternal gravida, birth weight and term between two groups. However, our findings showed that there was a larger proportion of new-borns delivered by SVD in the abnormal ocular group (*p* < 0.001) compared to new-borns with normal ocular findings.

We performed a subgroup analysis on new-borns delivered by SVD, as shown in Table 2. In terms of duration of 1st stage and 2nd stage of labour, there was no significant difference between 2 groups. However, we found that episiotomy correlated with the more abnormal ocular findings group compared to normal findings group in new-borns delivered by SVD (*p* = 0.023).

**Table 2:** SVD Characteristic of normal and abnormal ocular findings among healthy term new-borns in Hospital Kuala Lumpur

	Normal Ocular Findings		Abnormal Ocular Findings		***P*** value
	***N*** = 64		***N*** = 52		
	Mean (SD)	n (%)	Mean (SD)	n (%)	
**Duration of 1st Stage of Labour (minutes)**	395.45 (181.29)		381.49 (174.83) ^θ^		0.678 ^a^
**Duration of 2nd Stage of Labour (minutes)**	8.30 (10.41)		11.65 (8.72) ^¶^		0.068 ^a^
**Episiotomy**					
Yes		27 (42.2)		33 (63.5)	0.023 ^b^
No		37 (57.8)		19 (36.5)	

θ, ¶ Missing data =1

^a^Student’s t test

^b^Pearson’s Chi-Square test

Table 3 showed the type of ocular abnormalities among healthy new-borns. There was a total of 14 abnormalities found in anterior segments and 64 abnormalities in posterior segments. Among all these abnormalities, the most frequent abnormality was retinal haemorrhage, which occurred in 93.8% of posterior segments.

**Table 3:** Type of ocular abnormalities among healthy term new-borns in Hospital Kuala Lumpur

	Abnormalities found	
	Number	Percentage
**Anterior Segments**	14	100.0
Iris nevus	1	7.1
Subconjunctival haemorrhage	13	92.9
**Posterior Segments**	64	100.0
Retinal haemorrhage	60	93.8
Vitreous haemorrhage	2	3.1
Congenital hypertrophy of RPE	2	3.1

Table 4 presents the eight possible associated factors of retinal haemorrhages in healthy new-borns. These factors were analysed using Simple Logistic Regression with no adjustment for other co-variates. Among all these factors, we found that the mode of delivery was associated with retinal haemorrhages in healthy new-borns (*p* < 0.001). New-borns delivered by SVD had 3.43 higher odds of having retinal haemorrhage compared to new-borns delivered by LSCS (OR = 3.43; 95% CI 1.73, 6.78; p < 0.001). Other factors showed no association with retinal haemorrhage in new-borns.

**Table 4:** Associated risk factors of retinal haemorrhages among healthy term new-borns in Hospital Kuala Lumpur using Simple and Multiple Logistic Regression

Variables	Simple Logistic Regression			Multiple Logistic Regression^a^		
	b	Crude OR (95% CI)	p	b	Adjusted OR (95% CI)	p
**Newborns age (day of life)**	−0.02	0.98 (0.80, 1.21)	0.871			
**Birth weight (g)**	−0.00003	1.00 (0.999, 1.001)	0.937			
**Term**						
37 wks to 38 wks 6 days	0	1				
39 wks to 40 wks 6 days	0.08	1.08 (0.58, 2.02)	0.813			
41 wks to 41 wks 6 days	0.51	1.66 (0.27, 10.35)	0.589			
**Gender**						
Male	0	1				
Female	−0.12	0.89 (0.48, 1.64)	0.698			
**Race**						
Malay	0	1				
Chinese	0.87	2.39 (0.76, 7.51)	0.137			
Indian	0.69	1.99 (0.82, 4.82)	0.127			
Others	0.11	1.11 (0.21, 5.96)	0.899			
**Maternal age (year)**	−0.01	0.99 (0.95, 1.04)	0.778			
**Maternal gravida**						
One	0	1				
Two	−0.38	0.69 (0.29, 1.64)	0.399			
Three	−0.26	0.77 (0.33, 1.82)	0.558			
Four	−0.05	0.95 (0.38, 2.40)	0.918			
Five or more	−0.31	0.74 (0.27, 2.04)	0.557			
**Mode of delivery**						
LSCS	0	1		0	1	
SVD	1.23	3.43 (1.73, 6.78)	< 0.001	1.23	3.43 (1.73, 6.78)	< 0.001

^a^Multiple Logistic Regression using Forward LR method was applied

Hosmer-Lemshow test (*p* = 0.958), classification table (overall correctly classified percentage = 72.9%) and Nagelkerke R^2^ (0.140) were applied to check the model fitness

Multiple logistic regression was used to assess whether these factors were independently related to retinal haemorrhages in healthy new-borns. After adjusting for all the co-variates, we hereby confirm that SVD remained the greatest risk factor which has nearly 3.5 times higher risk of new-borns having retinal haemorrhage compared to LSCS. Other factors were found no association with retinal haemorrhage using multiple logistic regression (*p* > 0.05).

We also performed a subgroup analysis on associated risk factors of retinal haemorrhages among healthy term new-borns delivered by SVD using Simple Logistic Regression (Table 5). We found that for every 1-min increment in the duration of 2nd stage of labour, there was a 6% chance more likely to have retinal haemorrhage (OR = 1.06; 95% CI 1.01, 1.11; *p* = 0.025). Besides, we found that SVD involving episiotomy had 2.5 times higher odds of associating with retinal haemorrhage in new-borns compared to SVD without episiotomy (OR = 2.50; 95% CI 1.16, 5.40; *p* = 0.020). However, after adjusting all the variables using Multiple Logistic Regression, only duration of 2nd stage of labour remained as associated risk factor for retinal haemorrhage in patients delivered by SVD.

**Table 5:** Subgroup analysis of associated risk factors of retinal haemorrhages among healthy term new-borns delivered by SVD

Variables	Simple Logistic Regression			Multiple Logistic Regression^a^		
	b	Crude OR (95% CI)	p	b	Adjusted OR (95% CI)	p
**Duration of 1st Stage of Labour (minutes)**	−0.0001	1.00 (0.998, 1.002)	0.924			
**Duration of 2nd Stage of Labour (minutes)**	0.06	1.06 (1.01, 1.11)	0.025	0.06	1.06 (1.01, 1.11)	0.025
**Episiotomy**						
No	0	1				
Yes	0.92	2.50 (1.16, 5.40)	0.020			

^a^Multiple Logistic Regression using Forward LR method was applied

Hosmer-Lemshow test (*p* = 0.05), classification table (overall correctly classified percentage = 65.2%) and Nagelkerke R^2^ (0.074) were applied to check the model fitness

## Discussion

**Fig. 1:**
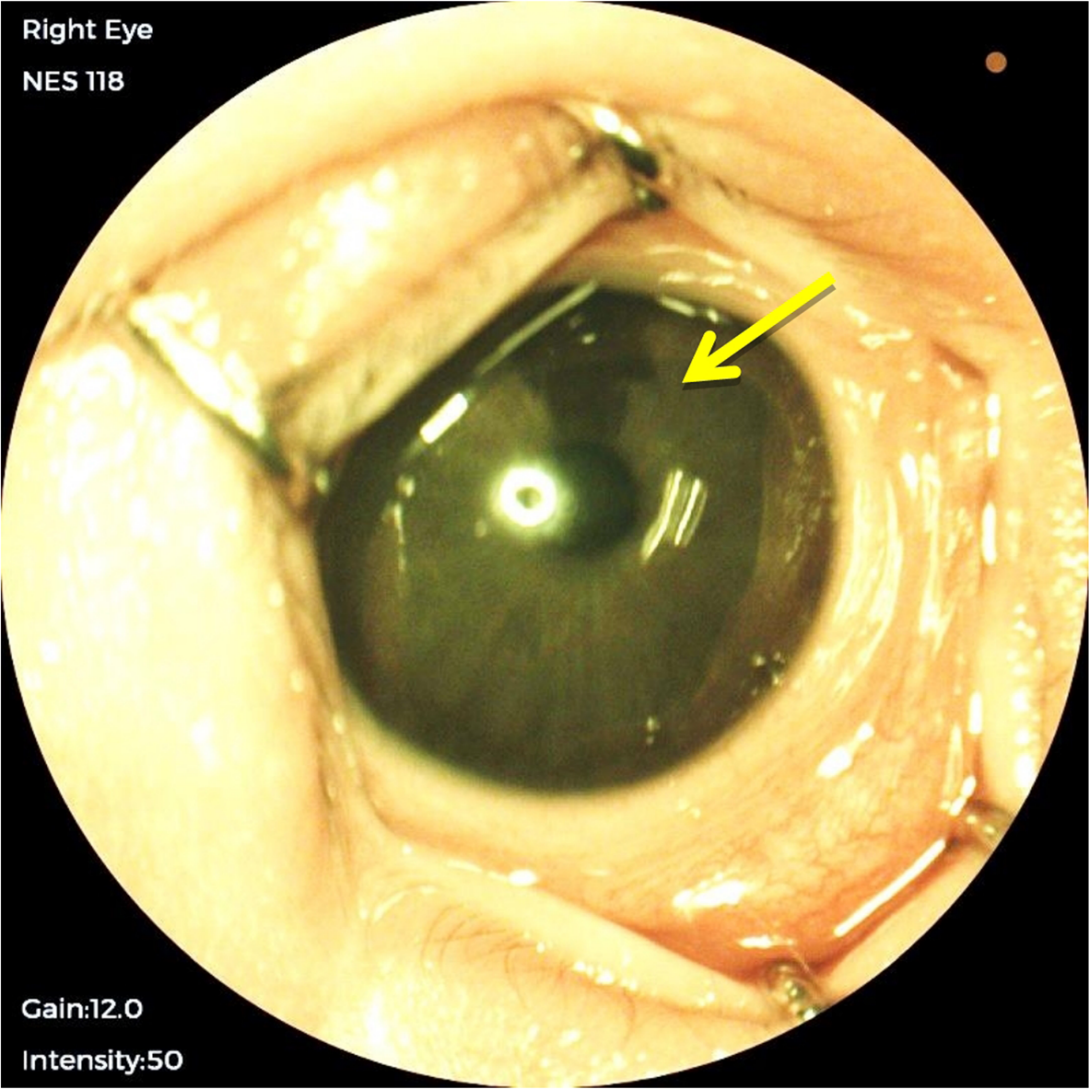
Right eye iris nevus (arrow)

**Fig. 2:**
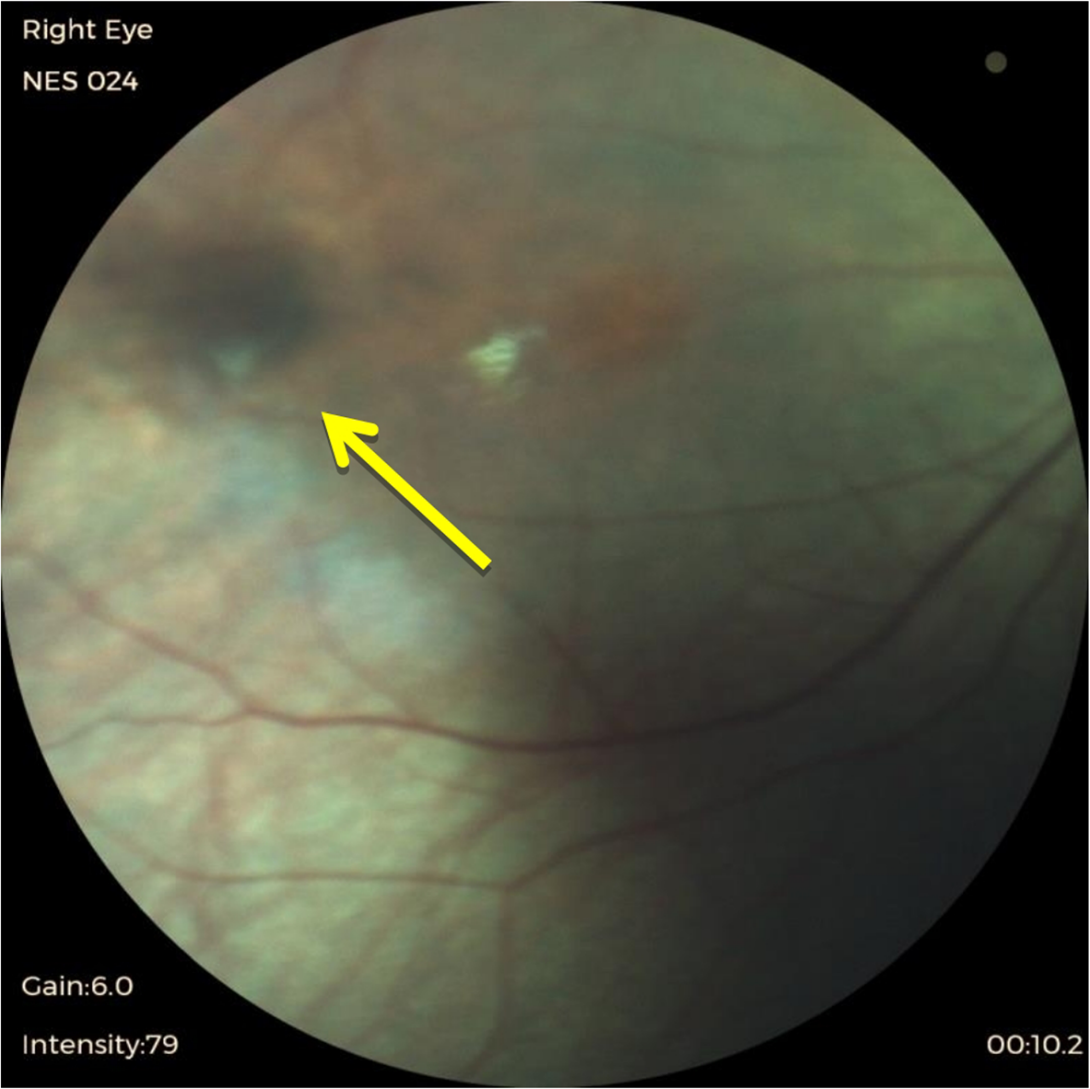
Congenital hypertrophy of retinal pigment epithelium (arrow)

**Fig. 3:**
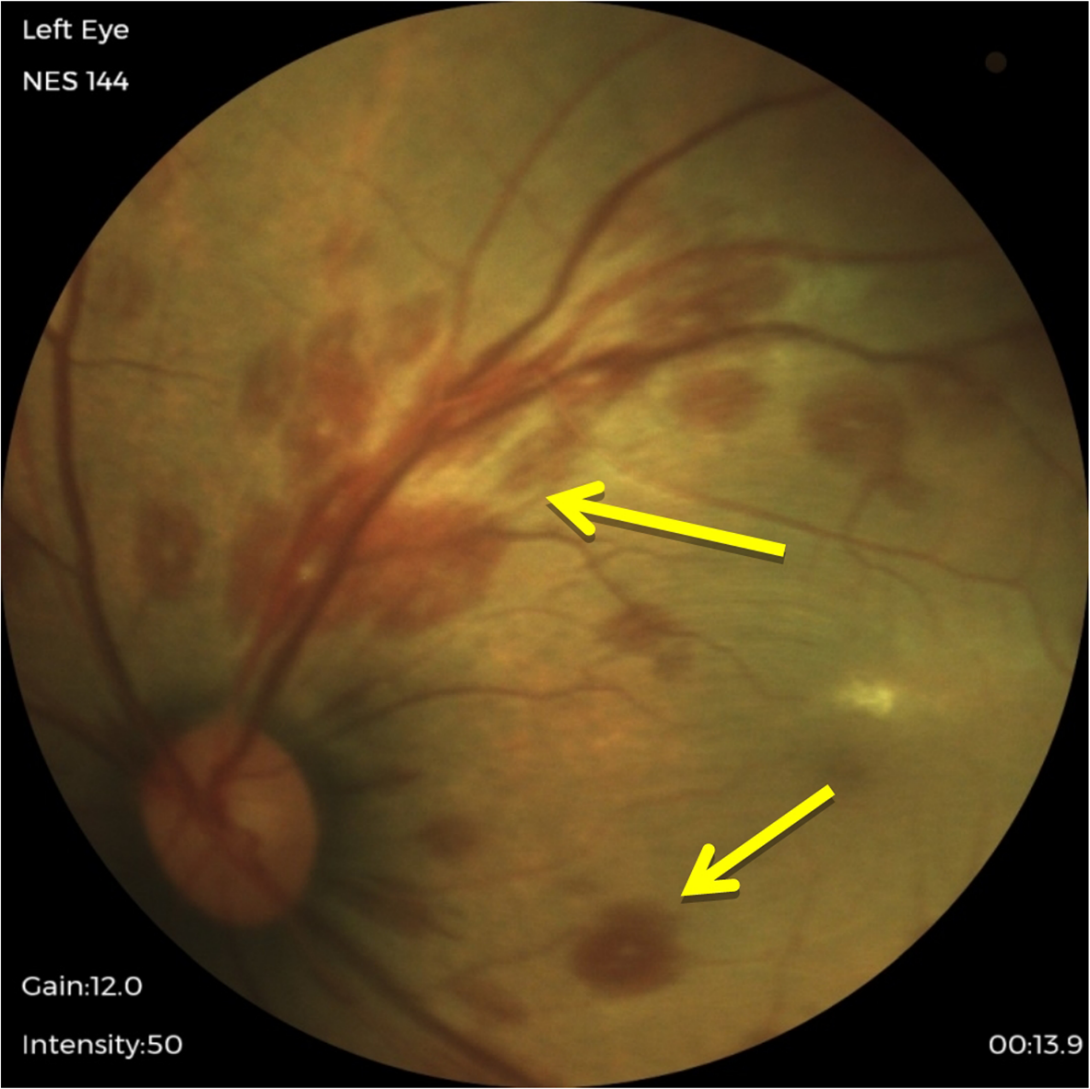
Retinal haemorrhages (arrow)

**Fig. 4:**
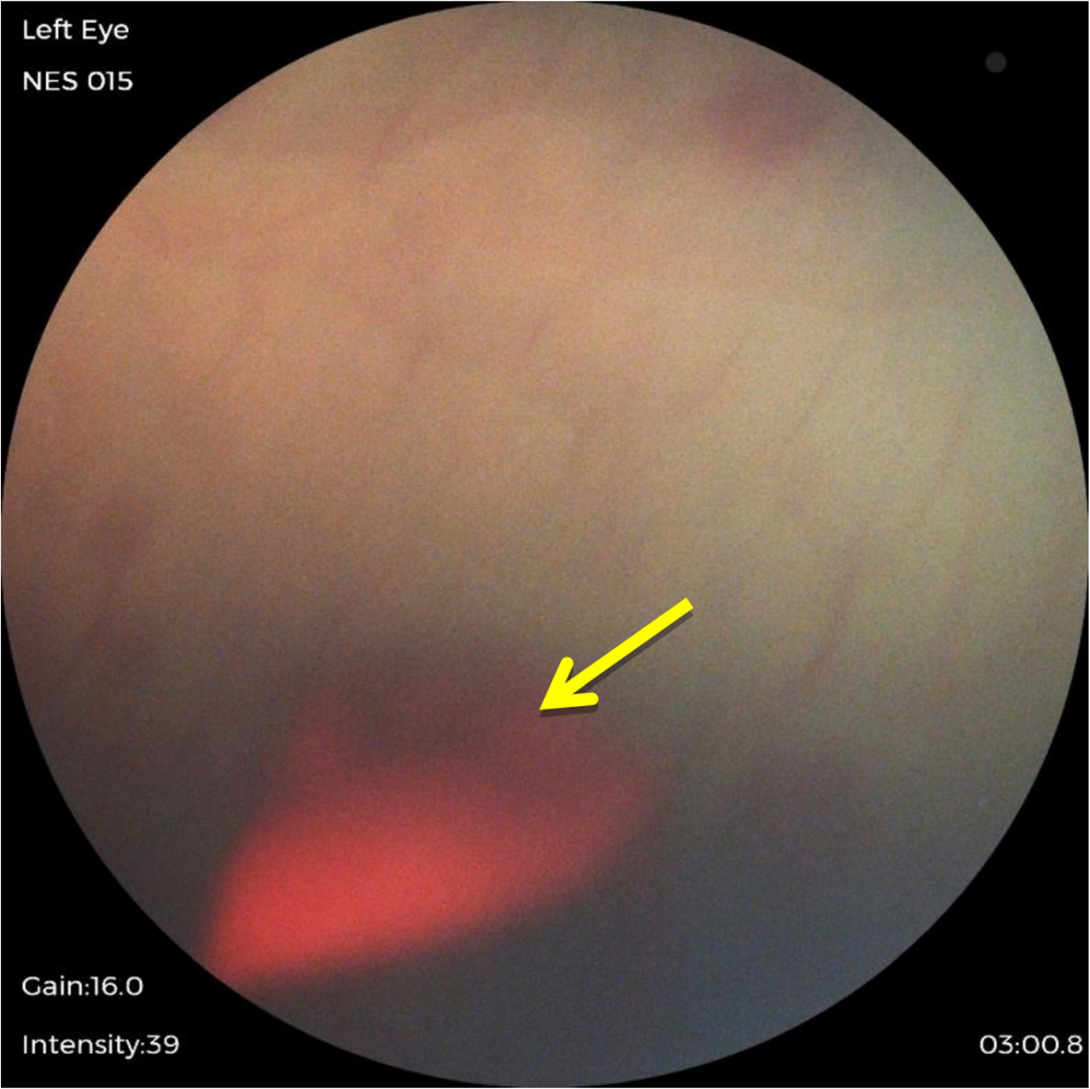
Vitreous haemorrhage (arrow)

The result of our study found that 34% of infants in the sample population had abnormal ocular findings, and it was higher than the expected range of 4 to 25% in other studies [2–4]. Ocular abnormalities detected in this study were subconjunctival haemorrhage, iris nevus (Fig. 1), congenital hypertrophy of retinal pigment epithelium (CHRPE) (Fig. 2), retinal haemorrhages (Fig. 3) and vitreous haemorrhage (Fig. 4). Compared to larger studies, we did not find any sight-threatening or life-threatening diseases such as congenital glaucoma, cataract and ocular tumours [2–4, 8, 11, 12]. This suggests that such diseases are infrequent at HKL. Nonetheless, they require urgent treatment if detected.

We evaluated some of the maternal, infant and birth factors which may contribute to risk for ocular abnormalities. However, we found no significant difference in gender, race, maternal gravida, birth weight and maturity of pregnancy between both groups, except there was a larger proportion of new-borns delivered by SVD in the abnormal ocular group (*p* < 0.001) compared to new-borns with normal ocular findings. We further sub-analyse the SVD group to compare 1st and 2nd stages of labour and episiotomy. In terms of duration of 1st stage and 2nd stage of labour, there was no significant difference between both groups. However, amongst new-borns delivered via SVD, we found that episiotomy was more common in the group with abnormal ocular findings compared to normal findings (*p* = 0.023).

In this study, the commonest abnormality was in the posterior segment, with 29.6% of infants having retinal haemorrhages. This result fell within the reported incidence of neonatal retinal haemorrhages between 2.6 to 50% [13]. The wide range of incidence reported may be due to sampling biases [14] and timing of examination [15]. Bilateral retinal haemorrhage was more common compared to unilateral (53.3% versus 46.6%), and this finding was similar to a systematic review by *Watts* et al. [15]. The incidences of retinal haemorrhages detected in this study were 47 and 45 cases in the right and left eyes respectively, suggesting that the probability of retinal haemorrhages was even for both eyes.

In recent years, many authors tried explaining mechanisms and risk factors for retinal haemorrhage and proposed that the likely cause was birth-related trauma [2, 16]. *Yanli* et al. proposed that compression of the foetal head during delivery causes deformation, which leads to elevated intracranial venous pressure, peripheral vascular congestion, expansion, or rupture, resulting in retinal haemorrhage [16]. So far, no studies have suggested otherwise.

Overall, there were no statistically significant findings in terms or gender, race, maternal gravida, birth weight, term and age of the infant when correlations were made to determine the risk factors for retinal haemorrhages. However, many studies have reported that the mode of delivery was an associated risk factor for retinal haemorrhage [8, 15, 16]. There are several other identified risk factors correlated to retinal haemorrhages such as maternal age, gravida, prolonged 2nd stage of labour and neonatal intracranial haemorrhage [16]. In our study, we looked into some of the risk factors reported in those studies. A multiple logistic regression analysis found indeed that SVD remained the greatest risk factor which has nearly 3.5 times higher risk of new-borns developing retinal haemorrhage compared to LSCS (OR = 3.43; 95% CI 1.73, 6.78; *p* < 0.001). A systematic review by *Watts* et al. found that instrument delivery had a higher risk compared to SVD [15, 17]. However, we did not compare the risk of assisted/instrumental delivery as there were only 5 cases of vacuum-assisted delivery (however 3 out of 5 cases were associated with retinal haemorrhage).

Subsequently, this study also found that for every 1-min increment in the duration of 2nd stage of labour, there was a 6% chance more likely to have retinal haemorrhage (OR = 1.06; 95% CI 1.01, 1.11; *p* = 0.025). *Yanli* et al. explained that during the second stage of labour, when the cervix is dilated fully, the foetal head will descend, and contractions become much stronger. At the same time, the foetus is affected by other stresses such as intrauterine ischaemia and hypoxia. Therefore, the longer the second stage of labour, the more serious the damage to the retinal vein and vascular endothelial cells, causing increased neonatal retinal haemorrhages [16].

At our study centre episiotomy is routinely performed on most primigravida mothers who deliver via SVD. This procedure involves a surgically planned incision on the perineum and the posterior vaginal wall during the second stage of labour. The aim is to enlarge the vaginal introitus to ease and facilitate safe delivery of the foetus, to minimise overstretching and rupture of the perineal muscles and fascia, to reduce the stress and strain on the foetal head, and to shorten the second stage of labour. A result in our study showed that SVD with episiotomy had 2.5 higher odds of associating retinal haemorrhage in new-borns compared SVD without episiotomy (OR = 2.50; 95% CI 1.16, 5.40; *p* = 0.020). However, it was statistically not significant after adjusting all the variables using Multiple Logistic Regression. *Emerson* et al also found that the incidence of retinal haemorrhages was not associated with episiotomy [13]. This suggest that episiotomy may even be protective against retinal haemorrhages as it is performed to reduce the duration of second stage of labour.

Many of these haemorrhages resolve spontaneously within 1–2 weeks without clinically significant long-term visual impairment [3, 15]. Some recover by 1 month [17]. Only a few of those cases required intervention [11]. Retinal haemorrhages rarely persist beyond 6 weeks, except in isolated cases, up to 58 days [15]. Therefore, the early detection of retinal haemorrhages can help distinguish between new-born ocular abnormalities and non-accidental injuries such as shaken baby syndromes [17]. *Choi* et al. reported a case of vitreous haemorrhage, which resolved after 3 months [18]. Some authors have postulated that long-standing; dense haemorrhage obscuring the macula may limit normal optical development, potentially resulting in visual disturbances such as anisometropia and amblyopia later in life [3, 18].

The ICON Paediatric fundus digital imaging system used in this study was able to produce clear images of the fundus for the detection and identification of posterior ocular abnormalities. Images taken are used in the form of visual education of parents. Many earlier studies reported using the Retcam. In some studies, these cameras were operated only by trained technician [2–4, 11, 12, 16, 19]. Therefore, examination using the fundus camera provided an alternative to indirect ophthalmoscopy by the Ophthalmologist. Technicians can be trained adequately to manage the Retcam cameras and subsequently stationed in places of high demand. This translates to better ophthalmologic services, simultaneously reducing the workload and address the issue of shortage of Ophthalmologist.

Evidence of earlier successful telemedicine programs for diabetic retinopathy and ROP suggests its extension to universal eye screening for all new-borns. Imaging of fundus abnormality allows us to record and monitor treatment and disease progression [20]. Images can also facilitate the transfer of information between clinicians [20]. Now, with the advent of 5G technology, examination and management of our patients can be done in real-time. However, the cost of purchasing these cameras is substantial, and its economic value has to be assessed further. A study by *Goyal P.* et al. attempted to prove that there was a net monetary gain when taken into consideration, for example, the potential financial loss incurred by treating a blind child [2].

In our study, there was no reported adverse effect from the screening procedure conducted on these healthy term new-borns. However, if present, previous studies showed that the systemic effects were mild and resolved spontaneously [1, 19, 21]. Early detection of eye diseases is important, and therefore, an eye examination ought to be done within 24-72 h after birth for all new-born unless they are unfit. If the new-born is unfit, the examination may be delayed. Further assessment by a family physician or general practitioner at 6 weeks of age is likely to enhance the detection rate further [6].

This study has several limitations. The convenient sampling method used, and relatively small sample size reduced the probability of detecting rarer ocular abnormalities. Multi-centre study would give a better representation of the true proportion and types of abnormalities detected in the new-born population.

## Conclusion

Universal eye screening for all new-borns using a wide-field digital imaging system is possible, safe and useful in detecting posterior segment disorders. The most common abnormality detected is retinal haemorrhage.

## Data Availability

The datasets used and/or analysed during the current study are available from the corresponding author on reasonable request.

## Abbreviations

BIO: Binocular indirect ophthalmoscope
CHRPE: Congenital hypertrophy of retinal pigment epithelium
HKL: Hospital Kuala Lumpur
HUKM: Hospital Universiti Kebangsaan Malaysia
LSCS: Lower segment caesarean section
MREC: Medical Research Ethics Committee
NMRR: National Medical Research Registry
O&G: Obstetrics and Gynaecology
REC: Research Ethics Committee
ROP: Retinopathy of prematurity
RPE: Retinal pigment epithelium
SVD: Spontaneous vaginal delivery
WFDRI: Wide-field digital retinal image
